# Unraveling the intra-species genomic diversity of sweetpotato-infecting CRESS-DNA and RNA viruses in Burkina Faso using Oxford Nanopore sequencing

**DOI:** 10.3389/fmicb.2026.1722370

**Published:** 2026-02-04

**Authors:** Pakyendou E. Name, Ezechiel B. Tibiri, Fidèle Tiendrébéogo, Seydou Sawadogo, Florencia Djigma, Lassina Traoré, Angela O. Eni, Justin S. Pita

**Affiliations:** 1 Laboratoire de Virologie et de Biotechnologies Végétales, Institut de l’Environnement et de Recherches Agricoles (INERA), Ouagadougou, Burkina Faso; 2 Laboratoire de Biologie Moléculaire et de Génétique (LABIOGENE), Université Joseph KI-ZERBO, Ouagadougou, Burkina Faso; 3 Central and West African Virus Epidemiology (WAVE), Pôle scientifique et d’innovation de Bingerville, Université Félix Houphouët-Boigny (UFHB), Bingerville, Côte d’Ivoire

**Keywords:** co-infections, CRESS-DNA viruses, deltasatellites, nanopore sequencing, sweetpotato, viral metagenomic

## Abstract

Sweetpotato is a key crop for global food security, particularly in Burkina Faso, where its productivity is increasingly threatened by viral diseases, especially those caused by CRESS-DNA viruses. However, the diversity of these viruses in Burkina Faso remains poorly characterized due to limitations of conventional diagnostic approaches. In this study, nanopore sequencing was used to investigate the diversity of CRESS-DNA viruses infecting sweetpotato in Burkina Faso. Ninety-eight symptomatic dried leaf samples from a previously established biobank were selected and analyzed. Total DNA was extracted, enriched using rolling circle amplification (RCA), and sequenced using the MinION Mk1C platform. In parallel, RNA viruses were also investigated using nanopore sequencing. RCA successfully amplified 53 of the 98 samples, from which 28 complete and 25 partial CRESS-DNA virus genomes were recovered. Sequence analyses revealed high genomic diversity, with sweet potato leaf curl virus (SPLCV) being the most prevalent. Sweet potato symptomless virus 1 (SPSMV-1) was detected for the first time in Burkina Faso in a co-infection with SPLCV. Additionally, 52 deltasatellite genomes (50 complete, 2 partial) were identified in association with SPLCV, displaying approximately 86% nucleotide identity with known sequences, suggesting the presence of genetically distinct putative deltasatellites. RNA virome analysis revealed frequent co-infections involving sweet potato feathery mottle virus (SPFMV) and sweet potato chlorotic stunt virus, with SPFMV commonly co-occurring with SPLCV. Four complete SPFMV genomes were recovered and clustered within phylogroup B, forming a distinct subclade. Overall, this study highlights the remarkable diversity of viruses infecting sweetpotato in Burkina Faso and reports, for the first time, the presence of SPSMV-1 and sweepovirus-associated deltasatellites in the country. These findings underscore the importance of ongoing molecular surveillance to support effective viral disease management strategies and food security.

## 1 Introduction

Sweetpotato (*Ipomoea batatas* [L.] Lam.) is a major food crop in tropical and subtropical regions (Mukhopadhyay et al., 2011; Okada et al., 2017), contributing substantially to food and nutritional security due to its high nutritional value, particularly in orange-fleshed cultivars rich in provitamin A (Somé et al., 2015; Kwak, 2019; Mwanga et al., 2021; Sapakhova et al., 2023). In Burkina Faso, it represents a strategic crop for small-scale farmers because of its high production (FAOSTAT, 2025), economic importance, and resilience to adverse climatic conditions (Somé et al., 2015; Tibiri et al., 2019a).

However, sweetpotato production is affected by abiotic and biotic constraints, particularly viral diseases. These viruses can have a significant impact on crop yield and quality, resulting in losses of up to 80% in some varieties (Adikini et al., 2016; Tibiri et al., 2019a; Wanjala et al., 2020). Of all the viruses identified to date, *Geminiviridae* is a significant family infecting sweetpotato worldwide (Trenado et al., 2011; Tibiri et al., 2020). This family of viruses is spread by whiteflies, leafhoppers, aphids, treehoppers, and can be distinguished genomically by their monopartite or bipartite single-stranded circular DNA (ssDNA) genomes (Fiallo-Olivé et al., 2021). Geminiviruses belong to a taxonomic group known as CRESS-DNA viruses (Circular Rep-Encoding Single-Stranded DNA, defined by the replication-associated protein (Rep) (Kazlauskas et al., 2019; Zhao et al., 2019; Krupovic et al., 2020). CRESS-DNA viruses are the most widespread and diverse characterized phytoviruses in ecosystems worldwide (Rosario et al., 2012; Zhao et al., 2019). Some studies have highlighted their wide host range, which includes plants, animals, humans, and various environmental reservoirs (Liu et al., 2020; Fehér et al., 2021; Zhao et al., 2021). CRESS-DNA viruses belong to the phylum *Cressdnaviricota*. This phylum comprises two classes (*Repensiviricetes* and *Arfiviricetes*), 13 orders, 24 families (including the family *Geminiviridae*), 269 genera and 1,560 species, all of which are officially recognized by the International Committee on Taxonomy of Viruses (ICTV).^1^

To date, two genera of the family *Geminiviridae* have been recorded on sweetpotato: *Begomovirus* and *Mastrevirus*. Begomoviruses, commonly known as sweepoviruses when infecting sweetpotato, are the most widespread sweetpotato viruses worldwide (Tibiri et al., 2019b). Sweet potato leaf curl virus (SPLCV), the prototypical sweepovirus, comprises 14 officially recognized species according to the International Committee on Taxonomy of Viruses (ICTV). SPLCV has been reported in Uganda (Wokorach et al., 2019), Tanzania (Bachwenkizi et al., 2022), Sudan (Langwa, 2019), Nigeria (Musa et al., 2021) and Burkina Faso (Tibiri et al., 2019b), and was recently identified in Benin (Chabi et al., 2024). The monopartite SPLCV genome, distinct from both Old and New World begomoviruses, contains six reading frames (ORFs), which is typical of monopartite begomoviruses (Fiallo-Olivé et al., 2021). Although the observed symptoms can be moderate, SPLCV infection can lead to significant reductions in production yield and economic losses (Trenado et al., 2011; Wanjala et al., 2020; Chabi et al., 2024).

Begomoviruses are known to interact with various satellites, including alphasatellites, betasatellites, and deltasatellites, which influence different stages of the viral infection process (Zhou, 2013). Previous studies on sweetpotato have characterized deltasatellites (the family *Tolecusatellitidae*) associated with sweepoviruses (Fiallo-Olivé et al., 2016; Hassan et al., 2016; Rosario et al., 2016; Ferro et al., 2021). These small, circular, single-stranded DNA molecules have no coding region and are believed to play a role in host-virus interactions (Hassan et al., 2016). Their genome is less than 1 kb in size and features a conserved stem-loop structure and a canonical nonanucleotide sequence (5’-TAATATTAC-3’) (Fiallo-Olivé et al., 2016). While their exact role in association with sweepoviruses remains unclear, recent studies suggest that they may influence symptom severity, thereby affecting disease dynamics (Hassan et al., 2016; Ferro et al., 2021).

Sweet potato symptomless virus 1 (SPSMV-1) is a member of the genus *Mastrevirus* found in sweetpotato. It was first identified in Peru (Kreuze et al., 2009), and has since been reported in China (Wang et al., 2015), Brazil (Souza et al., 2018), and Spain (Fiallo-Olivé et al., 2022). In Africa, it was first reported in Tanzania (Mbanzibwa et al., 2011) and in West Africa in Benin (Chabi et al., 2024). As its name suggests, symptoms in the event of infection are almost imperceptible but SPSMV-1 can contribute to yield losses through synergistic interactions with other viruses (Cao et al., 2017; Qiao et al., 2020).

Previous studies in Burkina Faso reported SPLCV in all major sweetpotato production regions (Tibiri et al., 2019b,^2020^). However, knowledge of the epidemiology and genomic diversity of CRESS-DNA viruses remains limited, and the emergence and increasing incidence of these viruses on crops in the sub-region are of concern (Ouattara et al., 2020). Conventional molecular detection methods have important limitations for identifying highly divergent or unexpected viruses. Recent advances in sequencing technologies, particularly Oxford Nanopore sequencing combined with rolling circle amplification (RCA), allow comprehensive characterization of viral genomes (Chehida et al., 2021; Aimone et al., 2022; Otron et al., 2025).

In this context, the present study aimed to characterize the diversity of CRESS-DNA viruses infecting sweetpotato in Burkina Faso, providing insights into their genomic diversity and epidemiology, which are critical for improving virus surveillance and management strategies.

## 2 Materials and methods

### 2.1 Targeted metagenomic approach to CRESS-DNA viruses

#### 2.1.1 Sample selection and total DNA extraction

**FIGURE 1:**
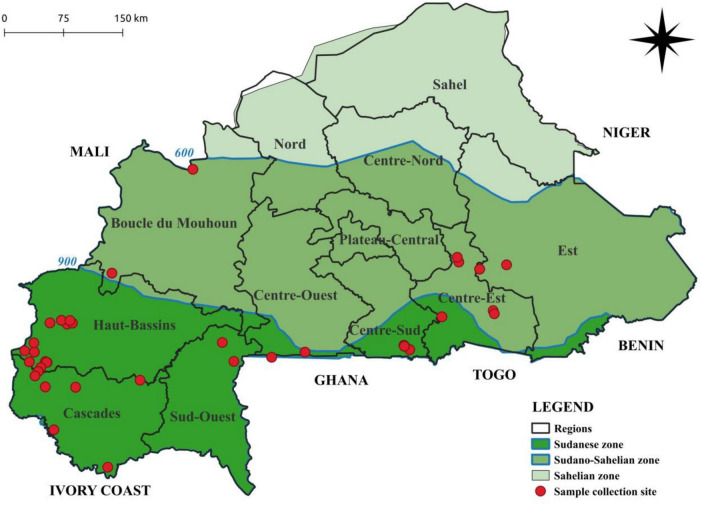
Map showing sample collection sites across Burkina Faso for this study.

A total of 98 symptomatic dried sweetpotato leaf samples were selected from a biobank of 600 available samples (Figure 1 and Supplementary Table 1) stored at the «Laboratoire de Virologie et de Biotechnologies Végétales (LVBV) » of INERA/CNRST, Burkina Faso. These samples were originally collected from eight major sweetpotato production regions across Burkina Faso between March 2016 and October 2017 (Tibiri et al., 2020). The samples included in this study were rigorously chosen to ensure optimal representativeness of these production areas within the biobank. Only samples with symptom scores between 2 and 9, as defined by the International Potato Center (CIP; McEwan et al., 2015) were included to ensure representation of relevant symptom diversity.

Total DNA was then extracted from dried leaves selected using the CTAB method as described by Doyle and Doyle (1987). DNA concentration and quality of the extracts were assessed using a NanoDrop 2000c UV-Vis spectrophotometer (Thermo Scientific, Wilmington, DE, USA), and extracts were stored overnight at 4°C, then at −20 °C for subsequent molecular analysis.

#### 2.1.2 Enrichment by rolling circle amplification (RCA)

The extracted total DNA was amplified using the TempliPhi DNA Polymerase Amplification Kit (Amersham Biosciences Corp.; Sunnyvale, CA, United States) in accordance with the protocol described by Inoue-Nagata et al. (2004). To achieve this, 1 μL of the total DNA (100 ng) was denatured with 5 μL of sample buffer at 95 °C for 3 min and then cooled directly on ice. A reaction mix consisting of 5 μL of reaction buffer and 0.2 μL of Phi29 DNA Polymerase Enzyme was then prepared and added. Amplification was carried out in a thermocycler at 30 °C for 20 h, followed by enzyme inactivation at 65 °C for 5 min.

#### 2.1.3 Library preparation and sequencing

The RCA products obtained were purified using Agencourt AMPure XP beads (Beckman Coulter, CA, USA, Cat. No. A63881). Next, 14 μL of the eluated DNA were digested using T7 endonuclease I (10 U) (New England Biolabs, MA, USA, Cat. No. M0302L) at 37 °C for 30 min, according to the protocol described by Chehida et al. (2021). This step was applied to resolve branched DNA structures generated during rolling circle amplification. Further purification was performed using Agencourt AMPure XP beads (Beckman Coulter, CA, USA, Cat. No. A63881) to remove residual enzyme and reaction components, ensuring clean DNA fragments suitable for downstream quantification and sequencing. The purified, digested products were quantified using a Qubit™ 4 fluorimeter (Thermo Fisher Scientific, Illkirch, France) with the Qubit™ dsDNA HS assay kit (Thermo Fisher Scientific, Illkirch, France, Cat. No. Q32854). The ends of the resulting DNA were then treated using the NEBNext Ultra II End Repair/dA-Tailing Module (New England Biolabs, MA, USA, Cat. No. E7546L), with an incubation period of 5 min at 20 °C, followed by 5 min at 65 °C. Following the purification of the end-prepped DNA, sequencing libraries were prepared using the Native Barcoding 96 V14 kit (SQK-NBD114.96, Oxford Nanopore Technologies, Oxford, UK), following the manufacturer’s instructions. Each sample was assigned a distinct barcode, allowing unambiguous identification of reads from each sample. Blunt/TA Ligase Master Mix (New England Biolabs, MA, USA, Cat. No. M0367L) was used to ligate the barcodes to the DNA fragments. After pooling all samples, adapter ligation was performed using NEBNext Quick Ligation Reaction Buffer (New England Biolabs, MA, USA, Cat. No. B6058S) and Quick T4 DNA Ligase (New England Biolabs, MA, USA, Cat. No. M2200S). The final libraries were loaded onto two Flow cells R10.4.1 (FLO-MIN114) and one Flongle Flow cell (FLO-FLG001). Sequencing was performed on a MinION device (Mk1C, Oxford Nanopore Technologies, Oxford, UK).

### 2.2 RNA virome-based metagenomic approach

#### 2.2.1 Sample selection

In this study, the frequent co-occurrence of circular single-stranded DNA viruses and RNA viruses in our sweetpotato samples justified the implementation of a complementary RNA-based approach to characterize their genomes and assess their role in mixed infections. Then, a total of 13 fresh sweetpotato leaf samples from the living germplasm of the biobank collection described earlier were taken from the INERA/CNRST experimental greenhouses. Fresh samples were preferred to preserve the integrity of the RNAs, which are naturally susceptible to degradation, thereby facilitating faithful RNA sequencing analyses.

#### 2.2.2 Extraction of total RNA and DNA removal

Total RNA extraction was performed using the Total RNA Purification Kit (NORGEN Biotek Corp., Ontario, Canada) according to the manufacturer’s instructions. The quality of extracts was assessed using a NanoDrop 2000c UV-Vis spectrophotometer (Thermo Scientific, Wilmington, DE, USA); and a Qubit™ 4 fluorimeter (Thermo Fisher Scientific, Illkirch, France) with the Qubit™ RNA HS Assay kit (Thermo Fisher Scientific, Illkirch, France, Cat. No. Q32855) and the Qubit™ RNA IQ Assay kit (Thermo Fisher Scientific, Illkirch, France, Cat. No. Q33221).

All traces of DNA were then removed by treating the RNA extracts with the RapidOut DNA Removal Kit (Thermo Fisher Scientific, Vilnius, Lithuania, Cat. No. K2981) according to the supplier’s instructions. The resulting DNA-free RNAs were transferred to a 1.5 mL Eppendorf LoBind tube previously placed on ice, the concentration was rechecked, and the RNA extracts were stored at −20 °C overnight and then at −80°C for further analysis.

#### 2.2.3 Ribodepletion

Ribodepletion is critical for optimizing transcriptome data, as it removes ribosomal RNA. It was performed using the QIAseq FastSelect -rRNA Plant Kit (QIAGEN, Hilden, Germany, Cat. No. 334315) according to the manufacturer’s instructions. A 40 μL mixture was prepared in a 0.2 mL PCR tube containing RNA (<2 μg), 8 μL of 5X First Strand Buffer, 1 μL of FastSelect reagent, and an adjusted volume of nuclease-free water. The mixture was subjected to a thermal program involving sequential decreases in temperature: 75 °C, 70 °C, 65 °C, 60 °C, 55 °C, 37 °C and 25 °C, with each temperature held for 2 min. Following this incubation, the ribodepleted RNA was purified using RNAClean XP Beads (Beckman Coulter, CA, USA, Cat. No. A63987), yielding enriched RNA samples.

#### 2.2.4 Library preparation and sequencing

Two library preparation protocols were optimized to improve sequencing efficiency and viral virome coverage. The first library, which was derived from ribodepletion products, was prepared using the Rapid Barcoding Kit 96 V14 (SQK-RBK114.96, Oxford Nanopore Technologies, Oxford, UK), following the manufacturer’s instructions and loaded onto a Flongle Flow cell (FLO-FLG001). Sequencing was then carried out using a MinION device MK1C, Oxford Nanopore Technologies, Oxford, UK). The second library was prepared using the cDNA-PCR Barcoding Kit 24 V14 (SQK-PCB114.24, Oxford Nanopore Technologies, Oxford, UK) with DNA-free RNA as the template, following the supplier’s instructions. This library was loaded onto a FLO-MIN114 Flow cell and sequencing was carried out using a MinION device (Mk1B, Oxford Nanopore Technologies, Oxford, UK).

### 2.3 Bioinformatic analysis workflow

The raw sequencing data, obtained in POD5 format, were basecalled in Super Accurate (SUP) mode using Dorado v0.9.0,^2^ which included adapter trimming and demultiplexing steps. All subsequent analyses, including quality assessment, reads cleaning, taxonomic classification, *de novo* assembly, polishing and annotation, were carried out using a, madehome pipeline that is currently under review for scientific publication. Although the pipeline is not publicly available at this time, it is documented at https://github.com/etibiri/denovo-assembly-pipeline.

### 2.4 Phylogenetic analysis and nucleotide identity matrix

The sequences obtained in this study were first analysed using the BLAST + 2.16.0 search tool^3^ against the NCBI database to identify closely related sequences. only complete genome sequences obtained in this study and reference sequences recognized by the ICTV were retained for subsequent comparative analyses (see Supplementary Table 2). Multiple sequence alignments were performed using ClustalW implemented in MEGA 11 (Tamura et al., 2021). Phylogenetic trees were then constructed in MEGA 11 using the maximum likelihood option, selecting the best-fit substitution model via the Bayesian Information Criterion (BIC) and all relevant parameters set within the software. Branch support was assessed using 1,000 ultra-fast bootstrap replicates (UFBoot). The resulting trees were visualized and annotated using FigTree v1.4.4.^4^

Species demarcation analyses was performed on complete genome sequences using SDT v1.2 (Muhire et al., 2014). Pairwise nucleotide identity matrices were generated using previously described datasets (see Supplementary Table 2). For begomoviruses, species and strain demarcation thresholds of ≥91% and ≥94% nucleotide identity, respectively, were applied (Brown et al., 2015). For deltasatellites, species were considered distinct at <91% nucleotide identity (Briddon et al., 2017). For potyviruses, species demarcation thresholds were applied following ICTV guidelines, including <76% nucleotide identity for the full-length open reading frame (ORF) and <76%–77% nucleotide identity for the coat protein, together with <80% amino acid identity (Adams et al., 2005).

## 3 Results

### 3.1 Occurrence and types of CRESS-DNA viruses

Following rolling circle amplification, successful amplification was observed in 53 out of 98 selected samples. These samples were distributed across the eight main sweetpotato production regions in Burkina Faso, ensuring broad geographical representativeness. After cleaning, taxonomic assignment of the viral reads revealed a high abundance of a limited number of CRESS-DNA virus taxa infecting sweetpotato in Burkina Faso. Of the nearly 1.26 million reads assigned to viruses, 840,000 belonged to the family *Geminivirhe* family *Geminidae*. Within this family, the genus *Begomovirus* was represented by SPLCV, while the genus *Mastrevirus* was represented by SPSMV-1.

Sweet potato leaf curl virus was identified in all analysed samples, with a total of 642,000 reads. SPSMV-1 was identified in a single sample from the Sud-Ouest region in association with SPLCV, with 246 reads assigned. The analysis also identified associated satellite DNA, particularly from the family *Tolecusatellitidae* (with 405,000 reads assigned). The predominant satellite was sweet potato leaf curl deltasatellite 3 (SPLCD3) with 404,000 reads. These deltasatellites were identified in all 53 successfully amplified samples in association with SPLCV. These results demonstrate the diversity of viruses present in sweetpotato in Burkina Faso.

*De novo* assembly was used to characterize the complete and partial genomes of the identified viruses. A total of 28 complete and 25 partial SPLCV genomes were obtained from the 53 analysed samples, confirming the taxonomic assignment results. Additionally, 52 deltasatellite genomes were assembled: 50 complete and two partial. Furthermore, a partial SPSMV-1 genome was successfully reconstructed from two non-contiguous contigs measuring 943 and 445 bp, respectively. The complete sequences were considered in subsequent analyses. A detailed overview of the isolates, genome coverage and assembly parameters are provided in Supplementary Table 3. All the sequences generated from this study have been deposited in the GenBank.

### 3.2 Characterization of complete genome of CRESS-DNA viruses

The 28 SPLCV genomes obtained in this study ranged in size from 2,804 to 2,832 bp. BLASTn analyses showed varying levels of nucleotide sequence identity between our isolates and SPLCV reference sequences listed by the ICTV.

BLASTn analyses showed that the SPLCV isolates recovered in this study were closely related to previously described reference genomes, with nucleotide identities ranging from 94.3% to 97.7%. Most isolates were most closely related to the Burkina Faso isolate SPLCV-BFA43, while others showed highest similarity to reference isolates from China, the USA, and South Korea. Detailed BLASTn comparisons, including genome sizes and nucleotide identity values, are provided in Table 1.

**TABLE 1:** BLASTn-based comparison of SPLCV isolates identified in this study.

Sample	Accession number	Genome size (bp)	Closest reference isolate	Accession number	Country	Nucleotide sequence identity (%)	Query coverage (%)
Kan_BFA160	PV947737	2828	SPLCV-BFA43	LS991864	Burkina Faso	97.07	100
Tie_BFA266	PV947750	2828	SPLCV-JS	FJ176701	China	96.63	100
Tie_BFA310	PV947753	2825	SPLCV-GS	MH388496	South Korea	94.90	100
Tie_BFA313	PV947754	2829	SPLCV-I	OR866203	Trinidad and Tobago	95.72	100
Tie_BFA314	PV947755	2829	SPLCV-I	KT992061	South Korea	94.35	100
Bag_BFA362	PV947731	2828	SPLCV-JS	FJ176701	China	96.74	100
Sam_BFA608	PV947746	2828	SPLCV-BFA43	LS991864	Burkina Faso	97.46	100
Sam_BFA622	PV947747	2804	SPLCV-BFA43	LS991864	Burkina Faso	96.89	100
Kan_BFA641	PV947739	2827	SPLCV-BFA43	LS991864	Burkina Faso	97.60	100
Kan_BFA758	PV947742	2828	SPLCV-BFA43	LS991864	Burkina Faso	97.56	100
Kan_BFA773	PV947743	2829	SPLCV-BFA43	LS991864	Burkina Faso	96.78	100
Ban_BFA1094	PV947732	2829	SPLCV-BFA43	LS991864	Burkina Faso	96.86	100
Sam_BFA150	PV947745	2829	SPLCV-BFA43	LS991864	Burkina Faso	97.42	100
Tie_BFA224	PV947748	2829	SPLCV-I	OR866203	Trinidad and Tobago	96.33	100
Tie_BFA255	PV947749	2828	SPLCV-JS	FJ176701	China	97.09	100
Tie_BFA301	PV947751	2827	SPLCV-GS	MH388496	South Korea	95.75	100
Tie_BFA308	PV947752	2829	SPLCV-JS	FJ176701	China	96.39	100
Bag_BFA334	PV947729	2828	SPLCV-MG	JF768740	China	96.64	100
Bag_BFA351	PV947730	2828	SPLCV-BFA43	LS991864	Burkina Faso	96.89	100
Di_BFA386	PV947735	2828	SPLCV-BFA43	LS991864	Burkina Faso	97.60	100
Leo_BFA440	PV947744	2829	SPLCV-I	KT992061	South Korea	95.94	100
Dia_BFA496	PV947734	2826	SPLCV	AB433787	Japan	91.72	100
Kan_BFA638	PV947738	2828	SPLCV-BFA43	LS991864	Burkina Faso	97.70	100
Kan_BFA662	PV947740	2828	SPLCV-BFA43	LS991864	Burkina Faso	97.39	100
Kan_BFA692	PV947741	2828	SPLCV-BFA43	LS991864	Burkina Faso	97.24	100
Dou_BFA1047	PV947736	2824	SPLCV-BFA43	LS991864	Burkina Faso	97.46	100
Dan_BFA1298	PV947733	2828	SPLCV-BFA43	LS991864	Burkina Faso	96.15	100
Tie_BFA316	PV947756	2829	SPLCV-MG	JF768740	China	97.13	100

Full genome annotation of our isolates confirmed the organization typical of single-partite begomoviruses: six conserved open reading frames (ORFs). A comparative analysis of these ORFs with the SPLCV-US reference genome (AF104036) is presented in Table 2. Iteron-like motifs characterized by the GGWGA consensus sequence were also identified. These motifs were found in four different locations: three in the positive strand orientation (designated I, II and III) and one in the complementary strand orientation (IV). These motifs are located in the intergenic region (IR), particularly near the AC1 ORF and the TATA box sequence (see Table 3).

**TABLE 2:** Annotation and comparative analysis of full-length SPLCV genomes: ORFs, amino acid composition, and intergenic regions (IR).

*Begomovirus*	N° Accession	Length	IR	V1		V2		C1		C2		C3		C4	
		nt	nt	nt	aa (% sim.)	nt	aa (% sim.)	nt	aa (% sim.)	nt	aa (% sim.)	nt	aa (% sim.)	nt	aa (% sim.)
Kan_BFA160	PV947737	2828	278	765	254 (98.43)	345	114 (97.68)	1095	364 (96.44)	447	148 (95.86)	435	144 (96.78)	258	85 (94.96)
Tie_BFA266	PV947750	2828	278	765	254 (96.21)	345	114 (98.26)	1095	364 (94.98)	447	148 (97.99)	435	144 (96.32)	258	85 (92.25)
Tie_BFA310	PV947753	2825	278	765	254 (95.16)	345	114 (95.65)	1092	363 (92.97)	444	147 (94.18)	435	144 (97.47)	258	85 (95.35)
Tie_BFA313	PV947754	2829	279	765	254 (96.47)	345	114 (98.26)	1095	364 (93.61)	447	148 (95.30)	435	144 (95.17)	258	85 (91.09)
Tie_BFA314	PV947755	2829	279	765	254 (94.97)	345	114 (93.04)	1095	364 (93.97)	447	148 (90.83)	435	144 (91.03)	258	85 (94.57)
Bag_BFA362	PV947731	2828	278	765	254 (96.73)	345	114 (98.26)	1095	364 (96.07)	447	148 (94.41)	435	144 (95.63)	258	85 (95.74)
Sam_BFA608	PV947746	2828	278	765	254 (98.30)	345	114 (96.23)	1095	364 (94.70)	447	148 (94.63)	435	144 (96.55)	258	85 (94.19)
Sam_BFA622	PV947747	2804	278	765	254 (98.69)	345	114 (96.81)	1071	356 (92.69)	447	148 (94.41)	435	144 (96.55)	258	85 (94.19)
Kan_BFA641	PV947739	2827	277	765	254 (98.56)	345	114 (97.39)	1095	364 (94.98)	447	148 (94.63)	435	144 (96.09)	258	85 (93.80)
Kan_BFA758	PV947742	2828	278	765	254 (98.17)	345	114 (96.52)	1095	364 (94.89)	447	148 (94.85)	435	144 (96.32)	258	85 (94.19)
Kan_BFA773	PV947743	2829	279	765	254 (97.65)	345	114 (96.81)	1095	364 (95.98)	447	148 (95.53)	435	144 (97.01)	258	85 (95.74)
Ban_BFA1094	PV947732	2829	279	765	254 (97.12)	345	114 (97.10)	1095	364 (96.16)	447	148 (95.53)	435	144 (96.78)	258	85 (95.74)
Sam_BFA150	PV947745	2829	279	765	254 (98.82)	345	114 (96.23)	1095	364 (95.16)	447	148 (95.75)	435	144 (97.70)	258	85 (94.19)
Tie_BFA224	PV947748	2829	279	765	254 (97.52)	345	114 (96.81)	1095	364 (94.52)	447	148 (96.20)	435	144 (96.55)	258	85 (92.64)
Tie_BFA255	PV947749	2828	278	765	254 (96.73)	345	114 (98.26)	1095	364 (95.89)	447	148 (97.54)	435	144 (97.01)	258	85 (93.41)
Tie_BFA301	PV947751	2827	277	765	254 (96.34)	345	114 (95.65)	1095	364 (95.62)	447	148 (93.51)	435	144 (94.71)	258	85 (93.02)
Tie_BFA308	PV947752	2829	279	765	254 (96.60)	345	114 (97.97)	1095	364 (93.97)	447	148 (97.99)	435	144 (97.01)	258	85 (93.80)
Bag_BFA334	PV947729	2828	278	765	254 (96.73)	345	114 (97.68)	1095	364 (95.62)	447	148 (97.09)	435	144 (95.40)	258	85 (94.19)
Bag_BFA351	PV947730	2828	278	765	254 (98.82)	345	114 (96.23)	1095	364 (94.70)	447	148 (94.63)	435	144 (97.24)	258	85 (93.02)
Di_BFA386	PV947735	2828	278	765	254 (98.95)	345	114 (96.52)	1095	364 (95.89)	447	148 (97.87)	435	144 (97.24)	258	85 (96.12)
Leo_BFA440	PV947744	2829	279	765	254 (96.08)	345	114 (96.81)	1095	364 (93.88)	447	148 (93.96)	435	144 (97.01)	258	85 (93.41)
Dia_BFA496	PV947734	2826	277	765	254 (92.29)	345	114 (95.65)	1088	362 (90.32)	444	147 (88.81)	441	146 (89.80)	258	85 (92.25)
Kan_BFA638	PV947738	2828	278	765	254 (98.56)	345	114 (95.94)	1095	364 (94.70)	447	148 (95.08)	435	144 (96.55)	258	85 (93.80)
Kan_BFA662	PV947740	2828	278	765	254 (98.56)	345	114 (96.52)	1095	364 (94.79)	447	148 (94.63)	435	144 (96.55)	258	85 (93.80)
Kan_BFA692	PV947741	2828	278	765	254 (99.08)	345	114 (99.13)	1095	364 (95.53)	447	148 (95.75)	435	144 (95.86)	258	85 (94.19)
Dou_BFA1047	PV947736	2824	277	765	254 (98.82)	345	114 (98.55)	1095	364 (95.53)	444	147 (95.30)	432	143 (96.78)	258	85 (94.19)
Dan_BFA1298	PV947733	2828	278	765	254 (97.78)	345	114 (96.81)	1095	364 (93.74)	447	148 (93.96)	435	144 (94.71)	258	85 (92.25)
Tie_BFA316	PV947756	2829	279	765	254 (97.65)	345	114 (97.97)	1095	364 (95.89)	447	148 (98.21)	435	144 (97.01)	258	85 (93.80)

aa, amino acids; nt, nucleotides.

**TABLE 3:** Functional analysis of SPLCV isolates: iterative elements, consensus sequences, and N-terminal Rep iteron-related domain (IRD).

Virus	Sample	N° accession	Iterative elements					Consensus	Rep IRD
			I	II	III	TATA box	IV		
SPLCV	Kan_BFA160	PV947737	ATTTGGAGA	ATTGGAGA	GGAGAC	TATATA	TCTCC	GGWGAC	MAPPKRFKIQA
SPLCV	Tie_BFA266	PV947750	ATTTGGAGA	ATTGGAGA	GGAGAC	TATATA	TCTCC	GGWGAC	MAPPKRFKIQA
SPLCV	Tie_BFA310	PV947753	ATTTGGAGA	ATTGGAGA	GGAGAC	TATATA	TCTCC	GGWGAC	MAPPKRFKIQA
SPLCV	Bag_BFA362	PV947731	ATTTGGAGA	ATTGGAGA	GGAGAC	TATATA	TCTCC	GGWGAC	MAPPKRFKIQA
SPLCV	Sam_BFA608	PV947746	ATTTGGAGA	ATTGGAGA	GGAGAC	TATATA	TCTCC	GGWGAC	MAPPKRFKIQA
SPLCV	Sam_BFA622	PV947747	ATTTGGAGA	ATTGGAGA	GGAGAC	TATATA	TCTCC	GGWGAC	MAPPKRFKIQA
SPLCV	Kan_BFA641	PV947739	ATTTGGAGA	ATTGGAGA	GGAGAC	TATATA	TCTCC	GGWGAC	MAPPKRFKIQA
SPLCV	Kan_BFA758	PV947742	ATTTGGAGA	ATTGGAGA	GGAGAC	TATATA	TCTCC	GGWGAC	MAPPKRFKIQA
SPLCV	Kan_BFA773	PV947743	ATTTGGAGA	ATTGGAGA	GGAGAC	TATATA	TCTCC	GGWGAC	MAPPKRFKIQA
SPLCV	Ban_BFA1094	PV947732	ATTTGGAGA	ATTGGAGA	GGAGAC	TATATA	TCTCC	GGWGAC	MAPPKRFKIQA
SPLCV	Sam_BFA150	PV947745	ATTTGGAGA	ATTGGAGA	GGAGAC	TATATA	TCTCC	GGWGAC	MAPPKRFKIQA
SPLCV	Tie_BFA255	PV947749	ATTTGGGGA	ATTGGAGA	GGAGAC	TATATA	TCTCC	GGWGAC	MAPPKRFKIQA
SPLCV	Tie_BFA301	PV947751	ATTTGGAGA	ATTGGAGA	GGAGAC	TATATA	TCTCC	GGWGAC	MAPPKRFKIQA
SPLCV	Tie_BFA308	PV947752	ATTTGGAGA	ATTGGAGA	GGAGAC	TATATA	TCTCC	GGWGAC	MAPPKRFKIQA
SPLCV	Bag_BFA334	PV947729	ATTTGGAGA	ATTGGAGA	GGAGAC	TATATA	TCTCC	GGWGAC	MAPPKRFKIQA
SPLCV	Bag_BFA351	PV947730	ATTTGGTGA	ATTGGTGA	GGTGAC	TATATA	TCACC	GGWGAC	MAPPKRFKIQA
SPLCV	Di_BFA386	PV947735	ATTTGGAGA	ATTGGAGA	GGAGAC	TATATA	TCTCC	GGWGAC	MAPPKRFKIQA
SPLCV	Leo_BFA440	PV947744	ATTTGGAGA	ATTGGAGA	GGAGAC	TATATA	TCTCC	GGWGAC	MAPPKRFKIQA
SPLCV	Dia_BFA496	PV947734	ATTTGGAGA	ATTGGAGA	GGAGAC	TATATA	TCTCC	GGWGAC	MAPPKRFKIQA
SPLCV	Kan_BFA638	PV947738	ATTTGGAGA	ATTGGAGA	GGAGAC	TATATA	TCTCC	GGWGAC	MAPPKRFKIQA
SPLCV	Kan_BFA662	PV947740	ATTTGGAGA	ATTGGAGA	GGAGAC	TATATA	TCTCC	GGWGAC	MAPPKRFKIQA
SPLCV	Kan_BFA692	PV947741	ATTTGGAGA	ATTGGAGA	GGAGAC	TATATA	TCTCC	GGWGAC	MAPPKRFKIQA
SPLCV	Dou_BFA1047	PV947736	ATTTGGAGA	ATTGGAGA	GGAGAC	TATATA	TCTCC	GGWGAC	MAPPKRFKIQA
SPLCV	Dan_BFA1298	PV947733	ATTTGGAGA	ATTGGAGA	GGAGAC	TATATA	TCTCC	GGWGAC	MAPPKRFKIQA
SPLCV	Tie_BFA316	PV947756	ATTTGGAGA	ATTGGAGA	GGAGAC	TATATA	TCTCC	GGWGAC	MAPPKRFKIQA
SPLCV	Tie_BFA224	PV947748	ATTTGGTGA	ATTGGTGA	GGTGAC	TTTATA	TCACC	GGWGAC	MAPPKRFKIQA
SPLCV	Tie_BFA313	PV947754	ATTTGGTGA	ATTGGTGA	GGTGAC	TTTATA	TCACC	GGWGAC	MAPPKRFKIQA
SPLCV	Tie_BFA314	PV947755	ATTTGGTGA	ATTGGTGA	GGTGAC	TTTATA	TCACC	GGWGAC	MAPPKRFKIQA

### 3.3 Phylogenetic analysis and species delimitation

**FIGURE 2:**
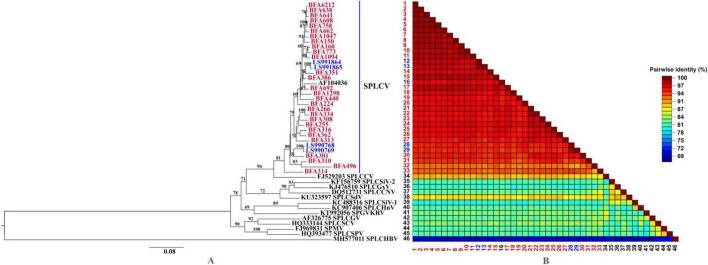
**(A)** Maximum likelihood phylogenetic tree of SPLCV isolates. Bootstrap values are given as percentages at the nodes. Sequences from this study are highlighted in red, SPLCV from Burkina Faso in blue and ICTV sequences in black. The tree was constructed using the Neighbor-Joining and BioNJ algorithms with a composite maximum likelihood (CML) method and the general time reversible (GTR) model with a discrete gamma distribution. **(B)** Pairwise sequence identity comparison matrix, showing identity values in the lower triangular matrix, sorted according to the phylogenetic tree.

The isolates obtained in this study (highlighted in red) and those previously reported from Burkina Faso (highlighted in blue) exhibited high pairwise nucleotide identity values, ranging from 95.0% to 99.6% based on complete-genome comparisons (Figure 2 and Supplementary Table 4). These values exceed the ICTV species demarcation threshold for begomoviruses (≥91%), as defined using all 14 ICTV-recognized SPLCV reference genomes. This supports their classification as SPLCV and excluding the presence of a novel SPLCV species in the analyzed dataset. Phylogenetic reconstruction further supported this classification, with all Burkina Faso isolates clustering within a well-supported phylogenetic clade that includes the SPLCV-US reference sequence (AF104036). This clustering indicates that the SPLCV population detected in Burkina Faso is composed of closely related isolates, consistent with previous reports of SPLCV circulation in the country.

However, two isolates, BFA496 (PV947734) and BFA314 (PV947755), clustered into a distinct subclade within the SPLCV group. This subclade was associated with slightly lower pairwise nucleotide identities (91%–93%) compared to other Burkina Faso isolates and reference sequences. These results indicate the presence of phylogenetically differentiated SPLCV variants circulating in Burkina Faso. Comparative analyses including global SPLCV and related sweepovirus sequences confirmed that all Burkina Faso isolates group within SPLCV and remain clearly separated from other sweepovirus species. Overall, these findings highlight the phylogenetic structuring and intraspecific variability of SPLCV isolates detected in Burkina Faso.

### 3.4 Discovery of deltasatellites associated with sweepoviruses

The analysis of CRESS-DNA virus diversity in sweetpotato samples resulted in the characterization of 50 complete and two partial deltasatellite genomes. These are single-stranded circular DNA molecules that are not CRESS and are associated with sweepoviruses. BLASTn analysis revealed that the 50 complete sequences exhibited 90%–92% nucleotide sequence identity and 88%–89% query coverage with the SPLCD3 isolate, which was identified in Puerto Rico (GenBank accession KT099179).

All assembled sequences exhibited the characteristic genomic organization of deltasatellites. In particular, all sequences contained the conserved nonanucleotide motif (5’-TAATATTAC-3’), which was located at the apex of a predicted stem-loop structure.

**FIGURE 3:**
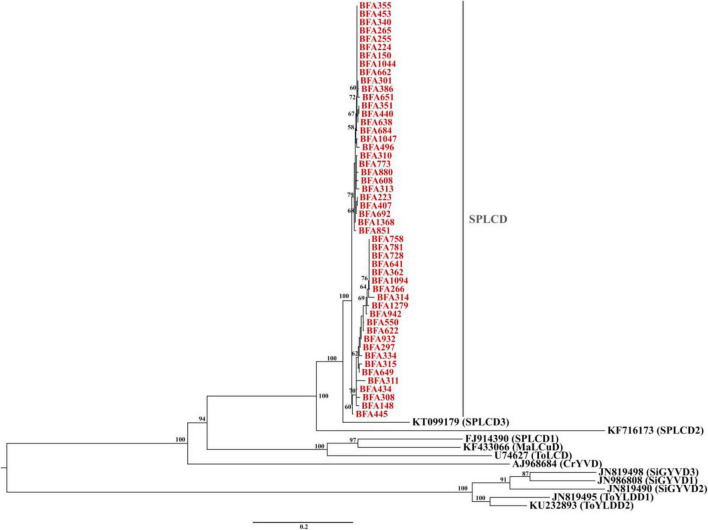
Maximum likelihood phylogenetic tree of SPLCD isolates. Bootstrap values are given as percentages and indicated at the tree nodes. Sequences from this study are highlighted in red, while ICTV sequences are highlighted in black. The tree was constructed using the Neighbor-Joining and BioNJ algorithms, based on a pairwise distance matrix estimated using Tamura’s 3-parameter model and a discrete gamma distribution with an invariant sites (I) model.

Phylogenetic analysis of the 50 isolates from Burkina Faso (Figure 3) showed that they cluster together into a well-supported phylogenetic clade. These sequences group closely with previously described sweepovirus-associated deltasatellites, particularly SPLCD1, SPLCD2 and SPLCD3, with high bootstrap support indicating robustness of the phylogenetic clustering. Pairwise nucleotide identity matrix analyses (see Supplementary Figure 1 and Supplementary Table 5) corroborated this phylogenetic structure, with identities among the Burkina Faso ranging from 94% to 100%, with an average of 97.8%. These values exceed the ICTV species demarcation threshold of 91% for members of the genus *Deltasatellite*. In contrast, comparisons with the SPLCD3 reference sequence (KT099179) showed lower nucleotide identity values, ranging from 83.6% to 86.7%. This indicates close relatedness but below the threshold required for assignment to the same species. Taken together, these results indicate that the deltasatellites isolates characterized in this study form a phylogenetically distinct clade within the genus *Deltasatellite*.

### 3.5 Virome profiling revealed the co-infections of CRESS-DNA viruses and RNA viruses

The RNA virome analysis of symptomatic sweetpotato leaf samples revealed very high viral diversity. The majority of viral reads (49,100) belonged to the family *Potyviridae* (48,300), with strong dominance of the genus *Potyvirus* (48,000 reads), which was mainly represented by the sweet potato feathery mottle virus (SPFMV). A small number of reads were assigned to the family *Closteroviridae* (genus *Crinivirus*, eight reads), which is represented by sweet potato chlorotic stunt virus (SPCSV). In parallel, CRESS-DNA viruses were detected in all 13 samples, With 706 reads assigned to the family *Geminiviridae* and genus *Begomovirus*, which was represented only by SPLCV. Traces of deltasatellites were also identified in some samples.

Among the analysed samples, single infections with SPFMV were observed in three samples (BFA214, BFA258, and BFA1311). Double infections (SPFMV + SPLCV) were the most frequent, observed in five samples (BFA297, BFA265, BFA880, BFA412, and BFA348). Triple infections involving SPFMV, SPLCV, and either SPCSV or deltasatellites were detected in three samples (BFA223, BFA728, and BFA423). These observations indicate that co-infections between RNA and CRESS-DNA viruses are common in the studied region, although the small sample size precludes generalizing to the broader sweetpotato population.

*De novo* assembly of the cleaned reads generated four complete SPFMV genomes (see Supplementary Table 3). Despite being clearly identified in some samples, the full SPCSV genome could not be reconstructed due to the shallow depth of the assigned viral reads.

### 3.6 Genomic analysis and overview of SPFMV isolates evolution

BLASTn analysis revealed that the four assembled SPFMV genomes shared nucleotide sequence identity with previously reported reference isolates. Isolate BFA297 (PV947757) showed 91.94% nucleotide identity with SPFMV-UNB-01 (MF185715) with 100% query coverage. Isolates BFA265 (PV947758) and BFA728 (PV947759) showed 91.10% and 89.76% nucleotide identity, respectively, with SPFMV 19-2036 (MT270302), with 100% query coverage in both cases. The isolate BFA880 (PV947760) showed 89.55% nucleotide identity with SSBles-111_ZA (MH023308) isolate with 100% query coverage.

Full annotation of our isolates revealed a genomic organization characteristic of potyviruses. The genomes range in size from 10,769 to 10,819 nucleotides and consist of a 5’ untranslated region (5’ UTR), followed by a single large open reading frame (ORF) that encodes a polyprotein. Two alternative reading frames resulting from transcriptional slippage are also present: P1N-PISPO, which is a known suppressor of RNA silencing, and P3N-PIPO, which is associated with viral movement.

A pairwise nucleotide identity matrix based on full-length open reading frames (ORFs) showed that the four SPFMV isolates sequenced in this study (PV947757-PV947760) shared 94.0%–100% nucleotide identity (Supplementary Table 6), indicating a high level of sequence similarity within this group. Comparisons with previously reported SPFMV isolates revealed nucleotide identities ranging from 90.2% to 98.6%. These values are well above the ICTV species demarcation threshold of 76% for potyviruses, thereby confirming their classification as SPFMV.

Although all isolates clustered clearly within the same species, lower nucleotide identity values (approximately 90%–92%) with some reference isolates indicate sequence differentiation among SPFMV isolates. Amino acid identity analyses showed a similar pattern, with values consistently exceeding 90% among SPFMV isolates, while the outgroup exhibited substantially lower identities (55.5%–72.1%), supporting its use as an external reference.

**FIGURE 4:**
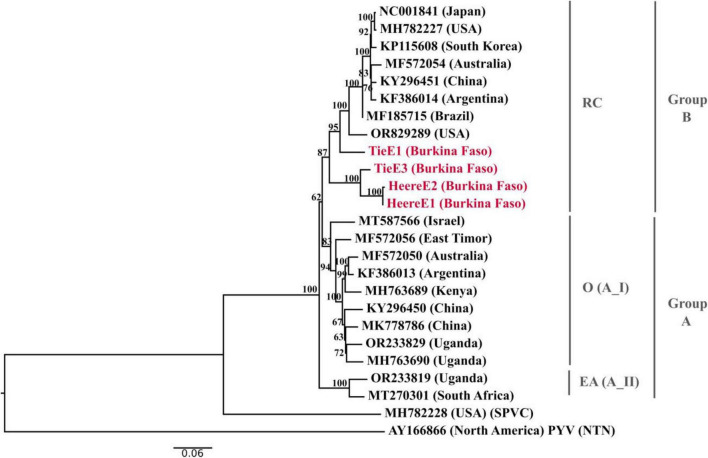
Maximum likelihood phylogenetic tree of SPFMV isolates. Bootstrap values (percentages) are shown at the nodes. The tree was inferred using a potato virus Y (PYV) isolate (AY166866) as a reference. Sequences from this study are highlighted in red and GenBank reference sequences are shown in black. The tree was constructed using the Neighbor-Joining and BioNJ algorithms, based on a pairwise distance matrix estimated using the Tamura-Nei model, incorporating a discrete gamma distribution and an invariant sites (I) model. The analysis included 25 sequences and the scale bar represents the number of nucleotide substitutions per site.

Phylogenetic analysis (Figure 4) corroborated these findings, showing that the four Burkina Faso isolates clustered within the major SPFMV clade corresponding to phylogroup B (RC strain). Within this phylogroup, the Burkina Faso isolates formed a distinct and well-supported phylogenetic subclade, consistent with the patterns observed in the nucleotide identity analyses.

Further analysis of the coat protein (CP) supported these observations. The CP gene of the four Burkina Faso isolates was 945 nucleotides in length and encoded a 315-amino-acid protein exhibiting the conserved structural features characteristic of potyviruses. Nucleotide identity comparisons with representative SPFMV isolates ranging from 93% to 99%, consistent with the values obtained for the full-length open reading frames (ORFs). The deduced amino acid sequences were highly conserved, and multiple sequence alignment confirmed the presence of the canonical DAG tripeptide motif in the N-terminal region of the CP in all four isolates. This motif, which is essential for aphid-mediated transmission in potyviruses, supports the functional integrity of the Burkina Faso SPFMV isolates.

## 4 Discussion

This study provides a characterization of sweetpotato-infecting CRESS-DNA viruses (SPLCV and SPSMV-1), sweepovirus-associated deltasatellites, and associated RNA viruses (SPFMV and SPCSV) in Burkina Faso. It highlights their intra-species genomic diversity and frequent co-occurrence patterns, with implications for viral epidemiology and crop management.

Sweet potato leaf curl virus was detected in all 53 successfully amplified samples across the eight main sweetpotato production regions of Burkina Faso, confirming its widespread distribution. These results are consistent with previous reports by Tibiri et al. (2020), which identified SPLCV as the predominant virus affecting sweetpotato in the country. The reconstruction of 28 complete and 25 partial genomes provides new data on the genetic composition of SPLCV populations circulating in Burkina Faso.

Pairwise nucleotide identity and phylogenetic analyses revealed that most of the isolates obtained in this study, as well as those previously characterized in Burkina Faso, cluster closely with the SPLCV-US reference genome (AF104036). The high nucleotide identity values observed within this group (95%–99.6%) supports their classification as SPLCV according to current ICTV species demarcation criteria (Fiallo-Olivé et al., 2021).

However, two isolates (BFA314 and BFA496) displayed slightly lower nucleotide identity values (91%–93%) relative to other Burkina Faso isolates and reference sequences, yet remained above the species demarcation threshold. These isolates formed a distinct and well-supported phylogenetic subcluster within SPLCV, indicating the presence of genetic variability within the species. The widespread occurrence of SPLCV may be favored by the abundance of its whitefly vector, *Bemisia tabaci* (Simmons et al., 2009; Romba et al., 2018), combined with vegetative propagation practices and the continuous circulation of infected planting material in sweetpotato production systems (Aimone et al., 2021; Nigam, 2021).

Sweet potato symptomless virus 1 was detected for the first time in Burkina Faso, representing the second report of this virus in West Africa its identification in Benin (Chabi et al., 2024). SPSMV-1 was detected in co-infection with SPLCV and deltasatellites. As a cryptic virus, SPSMV-1 is frequently asymptomatic, which can limit its detection using conventional diagnostic approaches (Yang and Rothman, 2004). Previous studies have show that, under mixed infection conditions, SPSMV-1 may influence disease expression or interact with other viruses (Fiallo-Olivé et al., 2022). Its detection highlights the relevance of metagenomics-based surveillance and underlines the need for further studies to access its prevalence and epidemiological significance in sweetpotato production systems in Burkina Faso.

Sweepovirus-associated deltasatellites were detected in all analysed samples, representing the first documented occurrence of these elements in Burkina Faso and, to our knowledge, in West Africa. A total of 50 complete and two partial genomes were reconstructed. Pairwise nucleotide identity analyses showed that these sequences share 83.6%–86.7% identity with the SPLCD3 reference sequence (KT099179), which is below the 91% species demarcation threshold currently applied to the genus *Deltasatellite* (Fiallo-Olivé et al., 2016; Lozano et al., 2016). Phylogenetic analyses indicated that the deltasatellite isolates from Burkina Faso cluster together in a well-supported phylogenetic group, distinct from previously described deltasatellites. Together with their conserved genome organization and the presence of characteristic nonanucleotide motifs, these features support their assignment as candidate deltasatellites within the genus. However, in accordance with ICTV guidelines, species demarcation cannot rely solely on sequence identity or phylogenetic clustering. In the absence of experimental validation, including targeted PCR amplification, Sanger sequencing, and biological assays assessing replication dynamics, helper virus interactions, and potential effects on host plants, the assignment of these isolates to a novel species remains tentative (Fiallo-Olivé et al., 2016; Ferro et al., 2021). Overall, these findings reveal previously undocumented genetic divergence and phylogenetic structuring among sweepovirus-associated deltasatellites in Burkina Faso. They also highlight the need for complementary molecular and biological investigations to better characterize their biological properties and their role in sweetpotato virus complexes.

The composition of CRESS-DNA viruses in sweetpotato is further influenced by their frequent co-infection with RNA viruses, particularly SPFMV and SPCSV. such mixed infections are well documented and are known to exacerbate symptom severity and cause significant yield losses (Adikini et al., 2016; Tibiri et al., 2019a). In line with these observations, our study examined the RNA virome to assess the complexity of viral associations and to characterize the types of co-infections occurring on sweetpotato in Burkina Faso.

The analysis revealed a range of co-infection patterns, ranging from double to triple infections. suggesting that sweetpotato may play a role as reservoirs for viruses. This is due to their vegetative propagation, which results in the long-term accumulation of viruses in local agrosystems (Kreuze et al., 2009). Among the RNA viruses, SPFMV was the most abundant, being present in all the samples analysed and always in association with SPLCV and SPLCD.

Despite their non-coding nature, the detection of delta satellites in RNA sequencing libraries might not reflect active coding expression, but rather a combination of host antiviral RNA interference (RNAi) responses and methodological artifacts from residual DNA. In plants, infection by DNA viruses and their satellites triggers an RNA interference (RNAi) antiviral response, generating virus-derived small interfering RNAs. Deep sequencing of these small RNAs enables the assembly and identification of complete viral and satellite genomes from RNA-seq data (Pooggin, 2018). Furthermore, the efficiency of DNase treatment, which is used to remove DNA prior to library preparation, is not absolute. Even low amounts of residual DNA may remain after digestion and contribute to sequences that are mapped to DNA elements in RNA-seq datasets (Li et al., 2022).

SPVD has been described as resulting from synergistic interactions between SPFMV and SPCSV (Clark et al., 2012; Zhang et al., 2020), leading to enhanced symptom severity and significant yield losses (Adikini et al., 2016; Tibiri et al., 2019a). However, SPCSV was less frequently detected in the present study and was always present in triple infections alongside SPFMV and SPLCV. These findings emphasize the prevalence of SPFMV in the sweetpotato virome of the studied region, suggesting complex viral interactions that could affect disease dynamics and management strategies. However, the relatively small sample size in this study may limit how widely these observations can be generalized, emphasizing the need for further research involving larger, more representative samples.

Complete characterization of the genome of four SPFMV isolates provided additional insights into the genetic variability of SPFMV circulating in the studied regions. All isolates clustered within phylogroup B (RC strain), and formed a closely related phylogenetic subgroup. While these results indicate a degree of genetic relatedness among the analyzed isolates, further sampling would be required to assess the full extend of SPFMV diversity in Burkina Faso. The conserved DAG motif within the coat protein supports the functional competence of aphid-mediated transmission and is consistent with previous reports on SPFMV transmission biology (Gadhave et al., 2020; Jiang et al., 2023).

This study further demonstrates the effectiveness of combining Oxford Nanopore Technologies with rolling circle amplification for the comprehensive detection and characterization of CRESS-DNA viruses infecting sweetpotato. This approach overcomes key limitations of earlier studies conducted in Burkina Faso which were based on conventional methods (Tibiri et al., 2019b,^2020^). While PCR-based approaches are limited for detecting unknown or unexpected viral agents (Yang and Rothman, 2004), the metagenomic strategy applied here enabled the reconstruction of complete viral genomes, including cryptic viruses and associated subviral elements such as deltasatellites and SPSMV-1. The ability to detect both DNA and RNA viruses, as well as resolve co-infections, with high accuracy confirms that ONT is a powerful alternative to routine diagnostics methods, particularly for complex and evolving viral communities. These results emphasize the importance of integrating these technologies into phytosanitary systems to promote early pathogen detection and better disease management.

## 5 Conclusion

This study provides a molecular characterization of sweetpotato DNA and RNA viruses in Burkina Faso using Oxford Nanopore sequencing, with a particular focus on intra-species genomic variation. The results confirm the dominance of SPLCV among CRESS-DNA viruses infecting sweetpotato across major production regions and document the first detection of SPSMV-1 in the country, extending its known geographical distribution.

A major contribution of this work is the identification and genomic characterization of sweepovirus-associated deltasatellites consistently detected alongside SPLCV. Although these elements show clear genetic divergence and form a distinct phylogenetic group relative to previously described deltasatellites, their taxonomic status remains tentative in the absence of experimental validation. This highlights an important limitation of the present study and underscores the need for future functional and biological investigations to clarify their replication dynamics, helper virus specificity, and potential impact on disease expression.

The frequent co-occurrence of SPLCV with RNA viruses, particularly SPFMV and occasionally SPCSV, reveals the complexity of viral assemblages affecting sweetpotato in Burkina Faso. These mixed infections suggest potential virus-virus interactions that may influence symptom development and epidemiological dynamics, warranting targeted experimental studies under local agroecological conditions.

Overall, this work demonstrates the value of Oxford Nanopore sequencing as an effective tool for uncovering viral diversity beyond the reach of conventional diagnostics. By providing a genomic baseline for major sweetpotato-associated viruses in Burkina Faso, this study lays the groundwork for future integrative research combining genomics, diagnostics, and epidemiology, with the ultimate goal of strengthening virus surveillance frameworks and supporting sustainable sweetpotato production systems in Burkina Faso.

## Data Availability

The raw data supporting the conclusions of this article will be made available by the authors, without undue reservation. The full genome sequences of the isolates obtained and reported in this paper have been deposited in the GenBank database (accession nos. PV947677–PV947760).

## Footnotes

1
https://ictv.global/taxonomy

2
https://github.com/nanoporetech/dorado

3
https://blast.ncbi.nlm.nih.gov

4
https://github.com/rambaut/figtree
