# Supplementary material for: Unraveling the intra-species genomic diversity of sweetpotato-infecting CRESS-DNA and RNA viruses in Burkina Faso using Oxford Nanopore sequencing
Source: Front Microbiol. 2026 Feb 4;17:1722370. doi: 10.3389/fmicb.2026.1722370 (PMC12913392; doi:10.3389/fmicb.2026.1722370)
Supplement: Supplementary file 1 [file Presentation_1.pptx]

## Slide 1
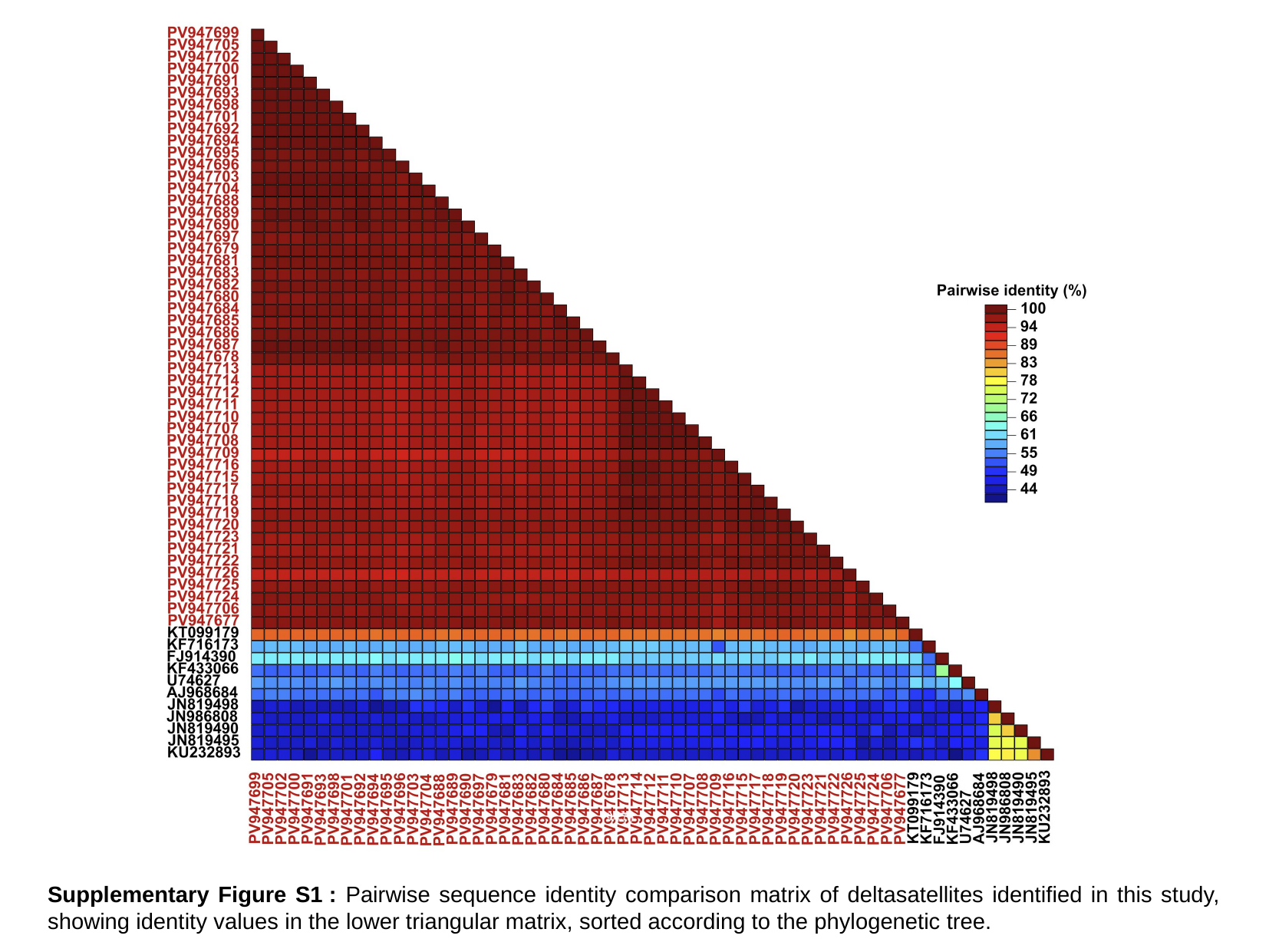

Supplementary Figure S1 : Pairwise sequence identity comparison matrix of deltasatellites identified in this study, showing identity values in the lower triangular matrix, sorted according to the phylogenetic tree.
