# Supplementary material for: Unraveling the intra-species genomic diversity of sweetpotato-infecting CRESS-DNA and RNA viruses in Burkina Faso using Oxford Nanopore sequencing
Source: Front Microbiol. 2026 Feb 4;17:1722370. doi: 10.3389/fmicb.2026.1722370 (PMC12913392; doi:10.3389/fmicb.2026.1722370)
Supplement: Supplementary file 2 [file Table_1.docx]

**Supplementary Table S1:** Geographical distribution and number of samples collected by region and district.

| **Regions** | **District** | **Number of samples selected** |
| --- | --- | --- |
| Boucle du Mouhoun | Di | 3 |
|  | Kouka | 2 |
|  | Tougan | 2 |
| Cascades | Banfora | 3 |
|  | Douna | 2 |
|  | Kankalaba | 3 |
|  | Mangodara | 1 |
|  | Niangologo | 1 |
|  | Sidéradougou | 1 |
| Centre-Est | Bagré | 5 |
|  | Koupéla | 1 |
|  | Ouargaye | 1 |
|  | Yargo | 2 |
|  | Yondé | 1 |
| Centre-Ouest | Boura | 2 |
|  | Léo | 6 |
| Centre-Sud | Tiebélé | 23 |
| Est | Diabo | 4 |
|  | Dianpagou | 1 |
| Hauts-Bassins | Kangala | 17 |
|  | Koloko | 2 |
|  | Samorogouan | 6 |
|  | Samogohiri | 7 |
| Sud-Ouest | Dano | 1 |
|  | Dissin | 1 |
