# Supplementary material for: Unraveling the intra-species genomic diversity of sweetpotato-infecting CRESS-DNA and RNA viruses in Burkina Faso using Oxford Nanopore sequencing
Source: Front Microbiol. 2026 Feb 4;17:1722370. doi: 10.3389/fmicb.2026.1722370 (PMC12913392; doi:10.3389/fmicb.2026.1722370)
Supplement: Supplementary file 3 [file Table_2.docx]

**Supplementary Table S2:** List of sequences retrieved from GenBank for identity and phylogenetic analyses.

| **Acronym** | **Isolates** | **Plant host** | **Location (Country)** | **N° Accession Genbank** |
| --- | --- | --- | --- | --- |
| **SPLCV** | | | | |
| SPLCV | BF:Bam:BFA43:16 | *Ipomoea batatas* | Bama (Burkina Faso) | LS991864 |
| SPLCV | BF:Leg:BFA181:16 | *Ipomoea batatas* | Leguema (Burkina Faso) | LS991865 |
| SPLCV | BF:Tie:BFA271:16 | *Ipomoea batatas* | Tiebele (Burkina Faso) | LS990768 |
| SPLCV | BF:TIe:BFA270:16 | *Ipomoea batatas* | Tiebele (Burkina Faso) | LS990769 |
| SPLCGV | US-Geo-16 | *Ipomoea batatas* | Georgia (USA) | AF326775 |
| SPLCHnV | CN-Hn10-12 | *Ipomoea batatas* | Henan (China) | KC907406 |
| SPLCSPV | BR-AlvM-09 | *Ipomoea batatas* | São Paulo (Brazil) | HQ393477 |
| SPLCSiV-1 | CN-Sc15-12 | *Ipomoea batatas* | Sichuan (China) | KC488316 |
| SPLCSiV-2 | CN-Sc14-12 | *Ipomoea batatas* | Sichuan (China) | KF156759 |
| SPLCSCV | US-SC-648-B9-06 | *Ipomoea batatas* | South Carolina (USA) | HQ333144 |
| SPLCV | US-Lou-94 | *Ipomoea batatas* | Louisiana (USA) | AF104036 |
| SPMV | BR-BSB1-08 | *Ipomoea batatas* | Brasilia (Brazil) | FJ969831 |
| SPLCCNV | CN: 05 | *Ipomoea batatas* | Liaoning (China) | DQ512731 |
| SPLCCV | ES:CI:BG25:02 | *Ipomoea batatas* | Canary Islands (Spain) | FJ529203 |
| SPLCGxV | CN-Gx5-11 | *Ipomoea batatas* | Guangxi (China) | KJ476510 |
| SPLCHbV | CN-Hb22-17 | *Ipomoea batatas* | Hubei (China) | MH577011 |
| SPGVKRV | KOR:20102:12 | *Ipomoea batatas* | South Korea | KT992056 |
| SPLCSdV | CN-SD-14 | *Ipomoea batatas* | Sao Paulo (Brazil) | KU323597 |
| **SPLCD** | | | | |
| CrYVD | IN-09 | *Croton bonplandianus* | Madurai (India) | AJ968684 |
| MaLCuD | PH-12 | *Malvastrum coromandelianum* | Philippines | KF433066 |
| SiGYVD1 | CU-177H1-09 | *Malvastrum coromandelianum* | Camaguey (Cuba) | JN986808 |
| SiGYVD2 | CU-228H1-09 | *Malvastrum coromandelianum* | Holguin (Cuba) | JN819490 |
| SiGYVD3 | CU-412N1-10 | *Malvastrum coromandelianum* | Matanzas (Cuba) | JN819498 |
| SPLCD1 | ES-SBG51-02 | *Ipomoea batatas* | Canary Islands (Spain) | FJ914390 |
| SPLCD2 | VE-1764E13-09 | *Merremia dissecta* | Sucre (Venezuela) | KF716173 |
| SPLCD3 | PR-T1_1-10 | *Bemisia tabaci/tomato* | Santa Isabel (Puerto Rico) | KT099179 |
| ToLCD | AU-96 | *Solanum lycopersicum* | Northern Territory (Australia AU) | U74627 |
| ToYLDD1 | CU-404N1-10 | *Sidastrum micranthum* | Matanzas (Cuba) | JN819495 |
| ToYLDD2 | CU-603N1-11 | *Sidastrum micranthum* | Matanzas (Cuba) | KU232893 |
| **SPFMV** | | | | |
| SPFMV | Bx_G0_11 | *Ipomoea batatas* | USA | OR829289 |
| SPFMV | Strain O, 24 | *Ipomoea batatas* | Bushenyi (Uganda) | OR233829 |
| SPFMV | Strain EA, 7 | *Ipomoea batatas* | Mpigi (Uganda) | OR233819 |
| SPFMC | Strain RC | *-* | Japan | NC001841 |
| SPFMV | - | *Ipomoea batatas* | Israel | MT587566 |
| SPFMV | 19-2035 | *Ipomoea batatas* | South Africa | MT270301 |
| SPFMV | Strain O, ji-17-49 | *Ipomoea batatas* | China | MK778786 |
| SPVC | Moyer-C | *Ipomoea nil* | USA | MH782228 |
| SPFMV | TFSW-1J | *Ipomoea nil* | USA | MH782227 |
| SPFMV | Ug-Lira2 | - | Uganda | MH763690 |
| SPFMV | Ke-Kirinya1 | - | Kenya | MH763689 |
| SPFMV | Strain O, TM66B | - | East Timor: Aileu | MF572056 |
| SPFMV | Strain RC, Aus9D | - | Broom (Australia) | MF572054 |
| SPFMV | Strain O, Aus13B | - | Fremantle (Australia) | MF572050 |
| SPFMV | UNB-01 | *Ipomoea batatas* | Pernambuco (Brazil) | MF185715 |
| SPFMV | Strain RC | *Ipomoea batatas* | China | KY296451 |
| SPFMV | Strain O | *Ipomoea batatas* | China | KY296450 |
| SPFMV | CW137 | *Ipomoea batatas* | South Korea | KP115608 |
| SPFMV | Strain RC | *Ipomoea batatas* | Argentina | KF386014 |
| SPFMV | Strain O | *Ipomoea batatas* | Argentina | KF386013 |
| PYV (NTN) | Tu_660 | *Solanum tuberosum* | North American | AY166866 |

SPLCV: sweet potato leaf curl virus; SPMV: sweet potato mosaic virus; SPLCCV: sweet potato leaf curl Canary virus; SPLCSiV-1: sweet potato leaf curl Sichuan virus 1; SPLCSiV-2: sweet potato leaf curl Sichuan virus 2; SPLCGxV: sweet potato leaf curl Guangxi virus; SPLCCNV: sweet potato leaf curl China virus; SPLCSdV: sweet potato leaf curl Shandong virus; SPLCHnV: sweet potato leaf curl Henan virus; SPGVKRV: sweet potato golden Korea vein virus; SPLCGV: sweet potato leaf curl Georgia virus; SPLCSPV: sweet potato leaf curl Sao Paulo virus; SPLCHBV: sweet potato leaf curl Hubei virus. SPLCD1: sweet potato leaf curl deltasatellite 1; SPLCD2: sweet potato leaf curl deltasatellite 2; SPLCD3: sweet potato leaf curl deltasatellite 3; MaLCuD: malvastrum leaf curl deltasatellite; ToLCD: tomato leaf curl deltasatellite; CrYVD: croton yellow vein deltasatellite; SiGYVD1: sida golden yellow vein deltasatellite 1; SiGYVD2: sida golden yellow vein deltasatellite 2; SiGYVD3: sida golden yellow vein deltasatellite 3; ToYLDD1: tomato yellow leaf distortion deltasatellite 1; ToYLDD2: tomato yellow leaf distortion deltasatellite 2; SPFMV: sweet potato feathery mottle virus; SPVC: sweet potato virus C
