# Supplementary material for: Unraveling the intra-species genomic diversity of sweetpotato-infecting CRESS-DNA and RNA viruses in Burkina Faso using Oxford Nanopore sequencing
Source: Front Microbiol. 2026 Feb 4;17:1722370. doi: 10.3389/fmicb.2026.1722370 (PMC12913392; doi:10.3389/fmicb.2026.1722370)
Supplement: Supplementary file 4 [file Table_3.docx]

**Supplementary Table S3:** Summary of complete viral genomes generated in this study, including accession numbers, coverage statistics, and assembly metrics.

| **Isolates** | **Virus** | **Accession N°** | **Length (bp)** | **% GC** | **Number of reads** | **% coverage** | **meandepth** | **meanbaseq** | **meanmapq** |
| --- | --- | --- | --- | --- | --- | --- | --- | --- | --- |
| Kan_BFA160 | SPLCV | PV947737 | 2828 | 45.3 | 808 | 100 | 70.2256 | 23.7 | 52 |
| Tie_BFA266 | SPLCV | PV947750 | 2828 | 44.9 | 13261 | 100 | 1567.94 | 24.3 | 55 |
| Tie_BFA310 | SPLCV | PV947753 | 2825 | 45.4 | 197121 | 100 | 25835.2 | 24.2 | 56 |
| Tie_BFA313 | SPLCV | PV947754 | 2829 | 44.8 | 22682 | 100 | 2303.75 | 23.9 | 53.4 |
| Tie_BFA314 | SPLCV | PV947755 | 2829 | 44.7 | 6291 | 100 | 989.018 | 24.3 | 52.4 |
| Bag_BFA362 | SPLCV | PV947731 | 2828 | 44.9 | 68997 | 100 | 5375.22 | 24 | 55.3 |
| Sam_BFA608 | SPLCV | PV947746 | 2828 | 45.6 | 23580 | 100 | 3098.2 | 24.6 | 53.5 |
| Sam_BFA622 | SPLCV | PV947747 | 2804 | 45.6 | 29106 | 100 | 4334.55 | 24.5 | 56.9 |
| Kan_BFA641 | SPLCV | PV947739 | 2827 | 45.6 | 42811 | 100 | 5124.87 | 24.3 | 52.6 |
| Kan_BFA758 | SPLCV | PV947742 | 2828 | 45.6 | 14734 | 100 | 1675.47 | 24.4 | 53.4 |
| Kan_BFA773 | SPLCV | PV947743 | 2829 | 45.2 | 4201 | 100 | 237.274 | 25.9 | 49.3 |
| Ban_BFA1094 | SPLCV | PV947732 | 2829 | 45.5 | 1112 | 100 | 116.772 | 22.4 | 51.7 |
| Sam_BFA150 | SPLCV | PV947745 | 2829 | 45.7 | 21781 | 100 | 1870.3 | 24.3 | 53.7 |
| Tie_BFA224 | SPLCV | PV947748 | 2829 | 45.6 | 36189 | 100 | 3505.55 | 24.1 | 56.2 |
| Tie_BFA255 | SPLCV | PV947749 | 2828 | 45.3 | 54965 | 100 | 5615.2 | 24.2 | 55.1 |
| Tie_BFA301 | SPLCV | PV947751 | 2827 | 45.3 | 125221 | 100 | 13758.2 | 24.5 | 53.9 |
| Tie_BFA308 | SPLCV | PV947752 | 2829 | 44.9 | 2676 | 100 | 307.372 | 24.3 | 51.5 |
| Bag_BFA334 | SPLCV | PV947729 | 2828 | 44.9 | 54253 | 100 | 4894.11 | 24.5 | 50.4 |
| Bag_BFA351 | SPLCV | PV947730 | 2828 | 45.2 | 12054 | 100 | 1502.4 | 24.4 | 47.3 |
| Di_BFA386 | SPLCV | PV947735 | 2828 | 45.7 | 4167 | 100 | 398.734 | 24.1 | 51.4 |
| Leo_BFA440 | SPLCV | PV947744 | 2829 | 45.4 | 63468 | 100 | 7231.58 | 24.1 | 53 |
| Dia_BFA496 | SPLCV | PV947734 | 2826 | 45 | 179592 | 96.0368 | 11780.6 | 23.6 | 47.8 |
| Kan_BFA638 | SPLCV | PV947738 | 2828 | 45.7 | 5804 | 100 | 647.046 | 24 | 54.2 |
| Kan_BFA662 | SPLCV | PV947740 | 2828 | 45.3 | 207263 | 100 | 18258.6 | 25.3 | 52.1 |
| Kan_BFA692 | SPLCV | PV947741 | 2828 | 45.2 | 2015 | 100 | 201.899 | 23.7 | 51.1 |
| Dou_BFA1047 | SPLCV | PV947736 | 2824 | 45.3 | 15219 | 100 | 1175.51 | 24 | 53.3 |
| Dan_BFA1298 | SPLCV | PV947733 | 2828 | 45.3 | 13101 | 100 | 1590.89 | 24.2 | 53.8 |
| Tie_BFA316 | SPLCV | PV947756 | 2829 | 45.3 | 108715 | 100 | 12612.5 | 19.1 | 48.9 |
| Sam_BFA148 | SPLCD | PV947706 | 707 | 47.7 | 291 | 100 | 207.536 | 23.8 | 58.8 |
| Kan_BFA160 | SPLCD | PV947727 | 628 | 47 | 189 | 100 | 128.623 | 23.3 | 58.4 |
| Tie_BFA266 | SPLCD | PV947708 | 703 | 46.9 | 1460 | 100 | 996.323 | 24 | 58.9 |
| Tie_BFA297 | SPLCD | PV947720 | 707 | 47.5 | 264 | 100 | 180.303 | 23.4 | 58.2 |
| Tie_BFA310 | SPLCD | PV947679 | 708 | 47.9 | 587 | 100 | 431.39 | 24 | 58.8 |
| Tie_BFA313 | SPLCD | PV947680 | 706 | 46.9 | 430 | 100 | 310.322 | 22.7 | 58 |
| Tie_BFA314 | SPLCD | PV947709 | 732 | 47.1 | 262884 | 100 | 164638 | 24 | 53.8 |
| Bag_BFA362 | SPLCD | PV947710 | 703 | 47.1 | 183 | 100 | 127.974 | 22.8 | 58 |
| Leo_BFA434 | SPLCD | PV947725 | 707 | 47.8 | 222 | 100 | 157.119 | 23.3 | 58.5 |
| Leo_BFA445 | SPLCD | PV947677 | 709 | 48 | 143 | 100 | 96.1721 | 23.3 | 58.8 |
| Sam_BFA550 | SPLCD | PV947717 | 704 | 47.6 | 228 | 100 | 156.655 | 21.5 | 59.1 |
| Sam_BFA608 | SPLCD | PV947682 | 708 | 47.6 | 181 | 100 | 119.527 | 24 | 59.3 |
| Sam_BFA622 | SPLCD | PV947718 | 704 | 47.6 | 232 | 100 | 158.418 | 22.3 | 59.3 |
| Kan_BFA641 | SPLCD | PV947711 | 703 | 47.1 | 202 | 100 | 139.232 | 23.7 | 59 |
| Kan_BFA728 | SPLCD | PV947712 | 703 | 47.2 | 297 | 100 | 212.75 | 23.1 | 57.8 |
| Kan_BFA758 | SPLCD | PV947713 | 703 | 47.2 | 288 | 100 | 214.132 | 22.5 | 58.7 |
| Kan_BFA773 | SPLCD | PV947681 | 707 | 47.4 | 364 | 100 | 252.115 | 23.5 | 58.2 |
| Kan_BFA781 | SPLCD | PV947714 | 703 | 47.2 | 265 | 100 | 194.312 | 23.5 | 58.9 |
| Kol_BFA851 | SPLCD | PV947678 | 707 | 47.8 | 181 | 100 | 123.209 | 23.5 | 59.1 |
| Sam_BFA880 | SPLCD | PV947683 | 705 | 47.4 | 145025 | 100 | 102042 | 23.7 | 58.6 |
| Tou_BFA932 | SPLCD | PV947719 | 704 | 47.6 | 212 | 100 | 153.298 | 23.8 | 58.3 |
| Sam_BFA942 | SPLCD | PV947715 | 704 | 46.9 | 431 | 100 | 316.3 | 22.9 | 58.4 |
| Ban_BFA1094 | SPLCD | PV947707 | 703 | 47.1 | 294 | 100 | 219.933 | 22.5 | 58.4 |
| Bou_BFA1279 | SPLCD | PV947716 | 703 | 47.5 | 230 | 100 | 148.319 | 23.3 | 58.4 |
| Sam_BFA150 | SPLCD | PV947698 | 707 | 48.1 | 324 | 100 | 227.785 | 22.9 | 58.6 |
| Tie_BFA223 | SPLCD | PV947684 | 705 | 48.2 | 397 | 100 | 282.692 | 22.4 | 58.3 |
| Tie_BFA224 | SPLCD | PV947693 | 707 | 47.9 | 512 | 100 | 344.405 | 22.8 | 58.2 |
| Tie_BFA255 | SPLCD | PV947691 | 707 | 47.9 | 311 | 100 | 216.969 | 23.9 | 58.7 |
| Tie_BFA265 | SPLCD | PV947700 | 706 | 48 | 564 | 100 | 409.809 | 22.9 | 58.7 |
| Tie_BFA301 | SPLCD | PV947694 | 715 | 48.1 | 344 | 100 | 240.544 | 23 | 58.5 |
| Tie_BFA308 | SPLCD | PV947724 | 707 | 47.5 | 170 | 100 | 121.468 | 22.3 | 58.3 |
| Tie_BFA311 | SPLCD | PV947726 | 703 | 47.2 | 11039 | 100 | 4069.65 | 24.2 | 57.4 |
| Tie_BFA315 | SPLCD | PV947721 | 702 | 47.3 | 213458 | 100 | 143600 | 24 | 59 |
| Bag_BFA334 | SPLCD | PV947723 | 704 | 47.2 | 612 | 100 | 383.143 | 23.4 | 58.2 |
| Bag_BFA340 | SPLCD | PV947702 | 705 | 48.1 | 567 | 100 | 385.711 | 23.3 | 58.3 |
| Bag_BFA351 | SPLCD | PV947703 | 704 | 47.7 | 281967 | 100 | 202435 | 23.5 | 58.3 |
| Bag_BFA355 | SPLCD | PV947699 | 707 | 47.9 | 831 | 100 | 593.758 | 22.9 | 58.5 |
| Di_BFA386 | SPLCD | PV947695 | 706 | 48.2 | 5743 | 100 | 4112.46 | 23.5 | 58.5 |
| Di_BFA407 | SPLCD | PV947685 | 705 | 48.4 | 201700 | 100 | 142737 | 23.7 | 58.7 |
| Leo_BFA440 | SPLCD | PV947704 | 705 | 47.8 | 807 | 100 | 581.559 | 23.1 | 58.8 |
| Leo_BFA453 | SPLCD | PV947705 | 705 | 48.1 | 351 | 100 | 252.607 | 23.5 | 59.2 |
| Dia_BFA496 | SPLCD | PV947697 | 707 | 47.7 | 197595 | 100 | 117264 | 23.6 | 58.6 |
| Kan_BFA638 | SPLCD | PV947688 | 706 | 47.7 | 398 | 100 | 271.596 | 23.4 | 59.2 |
| Kan_BFA649 | SPLCD | PV947722 | 703 | 47.7 | 325 | 100 | 228.526 | 22.6 | 57.7 |
| Kan_BFA651 | SPLCD | PV947696 | 707 | 48.5 | 856 | 100 | 584.461 | 23 | 58.3 |
| Kan_BFA662 | SPLCD | PV947692 | 705 | 48.1 | 596 | 100 | 402.976 | 23.1 | 57.9 |
| Kan_BFA684 | SPLCD | PV947689 | 719 | 47.8 | 881 | 100 | 600.049 | 23.2 | 58.8 |
| Kan_BFA692 | SPLCD | PV947686 | 707 | 47.9 | 442 | 100 | 316.207 | 22.9 | 58.3 |
| Dou_BFA1044 | SPLCD | PV947701 | 706 | 48 | 341 | 100 | 231.833 | 22.7 | 57.8 |
| Dou_BFA1047 | SPLCD | PV947690 | 707 | 47.8 | 492 | 100 | 329.017 | 23.1 | 58.6 |
| Dan_BFA1298 | SPLCD | PV947728 | 531 | 47.1 | 550 | 100 | 370.761 | 23.4 | 58.3 |
| Yon_BFA1368 | SPLCD | PV947687 | 707 | 47.8 | 256 | 100 | 169.047 | 22.8 | 59 |
| Tie_E1_BFA297 | SPFMV | PV947757 | 10816 | 41.9 | 11735 | 100 | 1730.44 | 38.8 | 59.6 |
| Tie_E3_BFA265 | SPFMV | PV947758 | 10797 | 41.9 | 5452 | 100 | 776.183 | 38.7 | 59.8 |
| Heere_E1_BFA728 | SPFMV | PV947759 | 10816 | 42.2 | 15635 | 100 | 2239.18 | 38.8 | 59.7 |
| Heere_E2_BFA880 | SPFMV | PV947760 | 10769 | 42.2 | 6078 | 100 | 801.17 | 38.9 | 59.8 |
